# AGING-RELATED CHANGES IN DAILY STRESSOR CONTROL AND GENERAL PERCEIVED CONTROL ACROSS THE LIFESPAN

**DOI:** 10.1093/geroni/igac059.479

**Published:** 2022-12-20

**Authors:** Eric Cerino, Susan Charles, Jacqueline Mogle, Jonathan Rush, Jennifer Piazza, Margie Lachman, David Almeida

**Affiliations:** Northern Arizona University, Flagstaff, Arizona, United States; University of California, Irvine, Irvine, California, United States; The Pennsylvania State University, University Park, Pennsylvania, United States; University of Victoria, Victoria, British Columbia, Canada; California State University, Fullerton, Fullerton, California, United States; Brandeis University, Waltham, Massachusetts, United States; The Pennsylvania State University, University Park, Pennsylvania, United States

## Abstract

Perceived control is an important psychosocial resource for healthy aging. Using data from the National Study of Daily Experiences (N=2,021, M=55.82 years, SD=10.35, 57.27% Female), we examined aging-related changes in stressor control across 10 years and compared these trajectories with measures of general control (mastery, constraints). Over 8 consecutive days in waves conducted in ~2008 and ~2017, people reported their perceived control over four types of stressors (arguments, avoided arguments, work, home). Longitudinal analyses revealed declines in stressor control across 10 years (p<.001), driven by declines in home stressors specifically. The rate of decline did not depend on baseline age. General control trajectories showed unique patterns of age differences in aging-related change such that declines (less mastery, more constraints) were steeper among older adults (p<.001). Results suggest that stressor control is a distinct domain of control beliefs with aging-related declines that differ based on type of stress experienced.

